# Serum Induces Transcription of Hey1 and Hey2 Genes by Alk1 but Not Notch Signaling in Endothelial Cells

**DOI:** 10.1371/journal.pone.0120547

**Published:** 2015-03-23

**Authors:** Kerstin Wöltje, Markus Jabs, Andreas Fischer

**Affiliations:** 1 Vascular Signaling and Cancer (A270), German Cancer Research Center (DKFZ-ZMBH Alliance), D-69120, Heidelberg, Germany; 2 Vascular Biology and Tumor Angiogenesis, Medical Faculty Mannheim (CBTM), Heidelberg University, D-68167, Mannheim, Germany; 3 Department of Medicine I and Clinical Chemistry, Heidelberg University, D-69120, Heidelberg, Germany; Feinberg Cardiovascular Research Institute, Northwestern University, UNITED STATES

## Abstract

The transcriptional repressors Hey1 and Hey2 are primary target genes of Notch signaling in the cardiovascular system and induction of *Hey* gene expression is often interpreted as activation of Notch signaling. Here we report that treatment of primary human endothelial cells with serum or fresh growth medium led to a strong wave of *Hey1* and *Hey2* transcription lasting for approximately three hours. Transcription of other Notch target genes (*Hes1*, *Hes5*, *ephrinB2*, *Dll4*) was however not induced by serum in endothelial cells. Gamma secretase inhibition or expression of dominant-negative MAML1 did not prevent the induction of *Hey* genes indicating that canonical Notch signaling is dispensable. Pretreatment with soluble BMP receptor Alk1, but not Alk3, abolished Hey gene induction by serum. Consequently, the Alk1 ligand BMP9 stimulated Hey gene induction in endothelial cells. Several other cell types however did not show such a strong BMP signaling and consequently only a very mild induction of *Hey* genes. Taken together, the experiments revealed that bone morphogenic proteins within the serum of cell culture medium are potent inducers of endothelial *Hey1* and *Hey2* gene expression within the first few hours after medium change.

## Introduction

*Hes* and *Hey* genes are the mammalian orthologes of *hairy* and *enhancer-of-split* genes in Drosophila.[1] They encode basic helix-loop-helix (bHLH) transcription factors that control expression of numerous loci in the human genome.[2] Both gene families are well known as the primary effector molecules of Notch signaling,[3] and both act predominantly as transcriptional repressors.[4]

The Delta-Notch cascade is a highly conserved and highly versatile signaling system that directs a multitude of binary and inductive cell fate decisions during development. Notch receptors (Notch1-Notch4) become activated upon binding of Delta-like or Jagged ligands (Jag1, Jag2, Dll1, Dll3, and Dll4) presented by adjacent cells. This leads to cleavage of the receptor and release of the Notch intracellular domain (NICD), which translocates to the nucleus. NICD interacts with Rbp-Jk (also known as CBF1 or Su(H)) that is bound to DNA and recruits MAML1 and transcriptional activators. Thereby, the Rbp-Jk complex is turned from a repressor into an activator of transcription.[3] This leads to the induction of a plethora of genes depending on the cell type and differentiation state.[4]

The most prominent Notch target genes are the *Hes* (*Hes1-Hes7*) and *Hey* (*Hey1*, *Hey2*, *HeyL*) transcription factors. At least one or two of these genes are activated by NICD in almost any cell type. Mouse knockout studies revealed that *Hes* genes execute Notch signals predominantly in the nervous, hematopoietic, endocrine and immune systems, while Hey genes are critically important in the cardiovascular system.[5]

Notch signaling is one of the few central regulators that guide almost every differentiation step during development, stem cell differentiation or regeneration.[6] Moreover, dysregulated Notch signaling occurs in several inherited syndromes as well as in cancer.[7, 8] Therefore, there is a great need to measure Notch signaling activity in cell culture and laboratory animals. This is not a trivial task. One well-accepted method is the detection of NICD by Western blotting,[9] which is technically challenging in cells with low Notch expression. Therefore, the indirect measurement of Notch target gene expression is most commonly used.

Expression of *Hey1* and *Hey2* in endothelial cells is dependent on Notch1 but both genes are still expressed in *Notch1* ^-/-^ mouse tissue, although at a lower level.[10] This indicates that other signaling pathways are involved in *Hey* gene expression, and indeed there is a complex crosstalk between Notch, TGF-beta and BMP signaling in endothelial cells, which allows a coordinated expression of *Hey* genes during angiogenesis.[11] Thereby, BMP signaling via Smad1/5 synergizes with Rbp-Jk to induce *Hey1* and *Hey2* expression.[12–15] Recent studies indicated that oscillatory expression of BMP-Smad and Notch target genes occurs in endothelial cells in a nonsynchronized manner. Thereby, individual endothelial cells become periodically competent to adopt either the tip cell or the stalk cell phenotype during angiogenesis.[13] Tip cells can guide new vessel sprouts whereas stalk cells follow and generate the lumen. However, this is not a static state and stalk cells actively take over the position and identity of a tip cell.[16] This behavior may be due to the oscillatory Notch and BMP-Smad1/5 loops in endothelial cells.[13] *Hes* and *Hey* gene expression occurs in an oscillatory manner in the presomitic and in neuronal progenitors.[17, 18] Likewise, *Hes1* expression was reported to oscillate upon stimulation with serum in several cultured cell types such as myoblastic and fibroblastic cell lines.[19] Interestingly, this process seems to be dependent of Stat and Smad signaling pathways.[20]

This study was inspired by the serendipitous observation that changing growth medium causes immense induction of *Hey1* and *Hey2* gene transcription in endothelial cells. The data demonstrate that at least in endothelial cells careful analysis of additional parameters is necessary to conclude that a certain treatment causes Notch signaling activation.

## Materials and Methods

### Plasmids, chemicals

cDNA encoding amino acids 13 to 74 of MAML1,[21] which encodes a dominant-negative form (provided by Jon C. Astor, Harvard) was cloned in frame with mCherry cDNA into pENTR3c and shuttled to pAd/CMV/V5 by Gateway cloning (LifeTechnologies). Adenoviral vectors were produced in HEK293 cells, and used at a MOI of 50. The gamma secretase inhibitor DAPT (N-[(3,5-difluorophenyl)acetyl]-L-alanyl-2-phenyl]glycine-1,1-dimethylethylester; Calbiochem) was used at 25 μM. The BMP type I receptor inhibitor LDN193189 (System Biosciences) was used at 3 μM. Cells were pretreated with DAPT or LDN193189 over night or for 30 minutes respectively. Recombinant human BMP9 and TGFβ1 were purchased from R&D systems and used at 10 ng/ml. Recombinant human Alk1 and Alk3 fused to human Fc were purchased from R&D systems and medium was preincubated for 30 minutes at a concentration of 5 μg/ml before adding to the cells.

### RNA isolation and qPCR analysis, Western blotting

RNA was purified with the RNeasy Mini Kit (Quiagen) and transcribed into cDNA (High Capacity cDNA Reverse Transcription Kit; Life Technologies). Real-time PCR was performed using the STEPOnePlus real-Time PCR system (Applied Biosystems). *RPS29* served as a house-keeping gene for normalization.

Protein lysates were subjected to SDS-PAGE blotted to nitrocellulose and incubated with antibodies against cleaved Notch1 (Abcam ab27526), pSmad1/5 (Ser463/465; Cell Signaling 9516), VCP (Abcam ab11433) or β-tubulin (Santa Cruz sc 9104) at 4°C overnight. After washing with TBST and incubation with peroxidase-coupled secondary antibodies, bands were detected with a chemiluminescence system (BioRad).

### Cell culture

HUVEC and HUAEC were grown and maintained until passage 5 in Endopan3 Growth Medium containing 3% FCS and supplements (Pan-Biotech). HBMVEC were maintained in BMEC growth media (PELO Biotech). HUASMC were cultured in high-glucose DMEM (Gibco) containing 15% FCS. HEK293, A549, and HeLa cells were cultured in DMEM with 10% FCS. FCS was from BioChrom and Sigma Aldrich, horse serum from BioChrom. HUVEC, HUAEC, HUASMC were freshly isolated, HBMVEC were purchased from PELO Biotech, Hela, A549 and HEK293T cells (ATCC numbers CCL-2, CCL-185, CRL-3216) were taken from the laboratory stock.

### Promoter analysis

Genomic sequences were obtained from NCBI, exons and translational start sites from ensembl.org. “rVISTA” was used with standard settings to find the Smad1 binding motif GCCGnCGC and to assess its conservation between different species.

### Statistical analysis

The results are presented as means + SD. Student’s t-test was used for pairwise comparisons between groups. p<0.05 was considered as statistically significant.

## Results

### Serum induces the expression of *Hey1* and *Hey2* in endothelial cells

**Fig 1:**
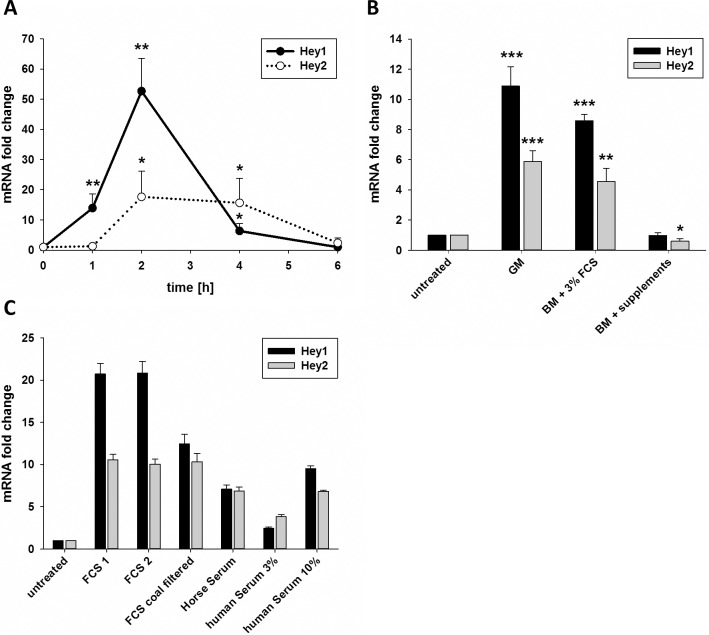
Serum induces transcription of *Hey1* and *Hey2*. A) HUVEC were grown in full growth medium and medium was changed to fresh growth medium at t0. The chart depicts the mRNA levels of *Hey1* and *Hey2*. B) HUVEC were grown in full growth medium and medium was changed either to fresh growth medium (GM), basal medium (BM) supplemented with 3% FCS or BM supplemented with a mix of growth factors (supplements). Transcript levels of *Hey1* and *Hey2* were determined after 1.5h and are shown in relation to samples with no medium change (untreated). C) HUVEC were grown in full growth medium and medium was changed to basal medium supplemented with 3% FCS purchased from two different suppliers, charcoal filtered FCS, horse serum or basal medium complemented with 3% or 10% human serum. Transcript levels of *Hey1* and *Hey2* are shown 1.5h after medium change. n = 3 (a,b) or one experiment shown (c). Mean and standard deviation of biological (a,b) or technical (c) replicates. Significant differences were calculated between the conditions marked with asterisks and t0 (a) or untreated control respectively.* p<0.05, ** p<0.01, *** p<0.001.

Induction of *Hey1* and *Hey2* transcription is often considered as readout for activation of Notch signaling in endothelial cells. We treated primary human umbilical venous endothelial cells (HUVEC) with different nutrients and metabolic compounds for two hours to screen for potential effects on Notch signaling. Surprisingly, this experiment suggested that all substances would largely induce *Hey1* and *Hey2* transcription. More careful analysis revealed however that the induction of *Hey1* and *Hey2* expression was due to the exchange of the endothelial growth medium only. The addition of fresh endothelial growth medium (Endopan3 with 3% FCS and supplements) after 24 hours culture led to a more than 10-fold induction of *Hey1* and *Hey2* mRNA expression in HUVEC. This induction could be detected after one hour, reached a maximum between 1.5 and 2 hours and lasted until 3 to 4 hours after the addition of fresh medium (**Fig. 1A**).

VEGF enhances *Hey* gene expression through induction of Dll4-Notch signaling.[22] VEGF is together with EGF, FGF, IGF, ascorbic acid, hydrocortisone and heparin a component of the endothelial growth medium supplement. However, VEGF alone or in combination with the other supplements was not responsible for the induction of *Hey* gene expression (**Fig. 1B**). Addition of basal medium (lacking FCS and supplements) did not alter Hey gene expression. However, the addition of 3% FCS led to almost the same induction of Hey gene transcription as the complete growth medium (**Fig. 1B**). The filtering of FCS with charcoal, which reduces the amount of lipophilic substances, did not significantly decrease the induction of *Hey1* and *Hey2* compared to non-filtered FCS (**Fig. 1C**). The importance of serum was further verified by treating HUVEC with other FCS preparations, as well as horse serum and serum from healthy adult human volunteers (**Fig. 1C**). As such, a hydrophilic serum compound was responsible for a short-term induction of *Hey* gene expression upon medium exchange.

Hey genes encode for transcriptional repressors, which orchestrate the expression of many other genes. In endothelial cells, *VEGFR2* and *VEGF* are important established target genes of Hey proteins. Given the time for Hey protein synthesis, we tested the expression of these genes four hours after adding serum to HUVEC. At this time point, mRNA levels of *VEGFR2* and *VEGF* were significantly down regulated to 67±13% and 75±11% respectively (n = 3; p < 0.05). This indicates that serum does not only induce *Hey* genes but indeed affects the regulatory network downstream of these transcriptional repressors as well.

### Serum-induced *Hey1* gene expression is independent of Notch signaling

**Fig 2:**
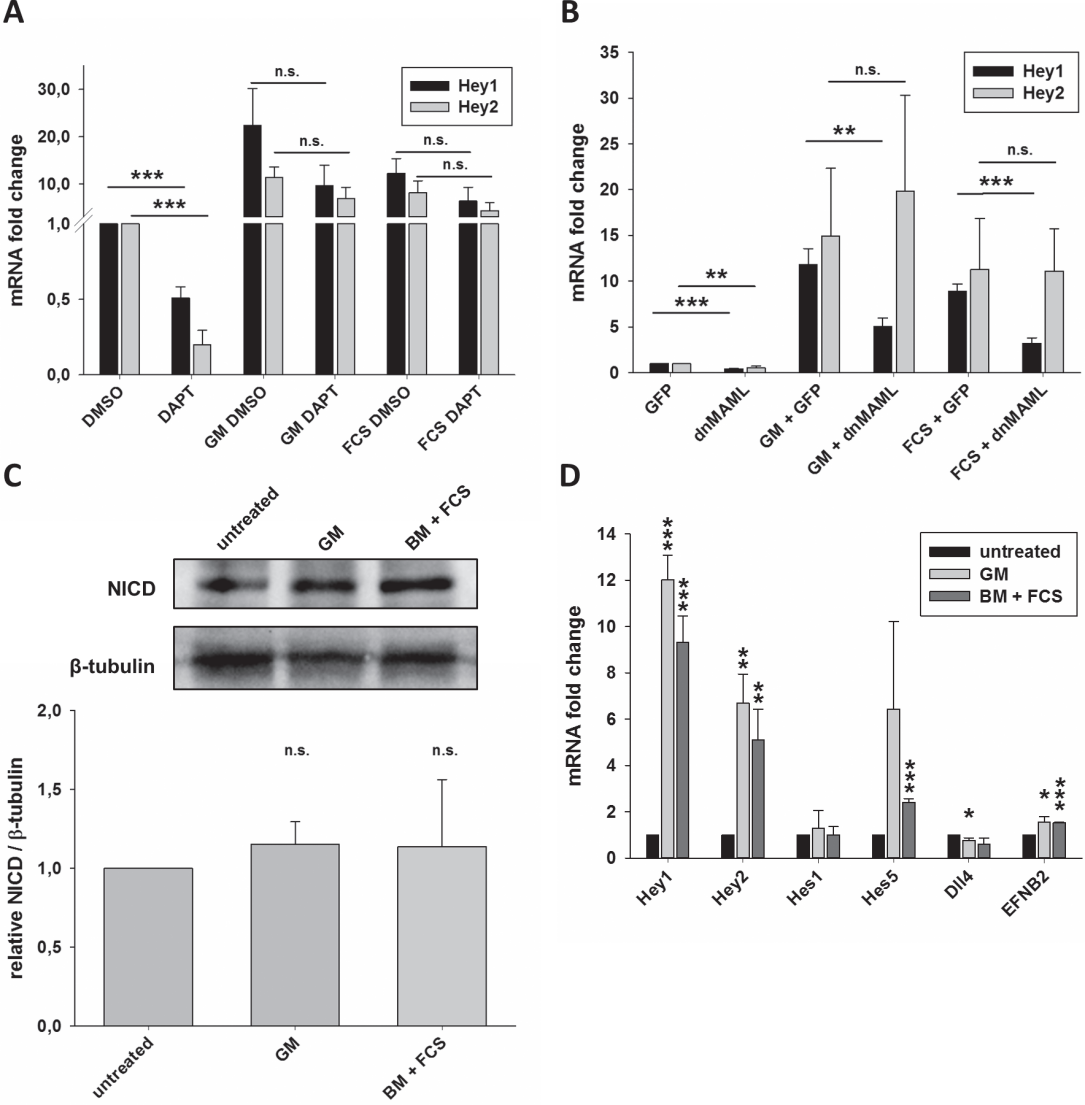
Induction of *Hey* genes by serum does not depend on Notch signaling. A) HUVEC were grown in growth medium containing 25μM DAPT or an equivalent volume of DMSO. Medium was unchanged (DMSO, DAPT) or changed to fresh growth medium (GM) or basal medium (BM) with 3% FCS containing DAPT or DMSO respectively. Transcript levels of *Hey1* and *Hey2* were measured 1.5h after medium change and are shown in relation to the sample incubated with DMSO, whose medium was not changed. B) HUVEC were transduced with adenoviral vectors expressing GFP or dnMAML. Cells were grown in growth medium. The medium was either unchanged (GFP, dnMAML) or replaced with fresh growth medium or basal medium containing 3% FCS. Transcript levels were determined after 1.5h and are normalized to the cells transduced with GFP and unchanged medium. C) HUAEC were grown in growth medium (untreated) or the medium was substituted with fresh growth medium or basal medium containing 3% FCS. The western blot is showing the cleaved intracellular domain of Notch1 (NICD) and β-tubulin 30 minutes after medium change. The chart summarizes four independent experiments and depicts the relative levels of NICD normalized for β-tubulin. D) HUVEC were left untreated or the medium was changed to fresh growth medium or basal medium supplemented with 3% FCS. After incubating 1.5h, the cells were harvested and the mRNA levels of several established Notch target genes (*Hey1*, *Hey2*, *Hes1*, *Hes5*, *Dll4 EFNB2*) were determined. Fold changes and significances were calculated to the untreated control. Mean and standard deviation, n = 3, * p<0.05, ** p<0.01, *** p<0.001, n.s. not significant.

*Hey* genes are regarded as the primary Notch target genes in endothelial cells.[10] Therefore, we tested if inhibition of Notch signaling activity would abolish the induction of *Hey1* and *Hey2* expression upon treatment with serum. HUVEC were pretreated with the gamma-secretase inhibitor DAPT overnight to inhibit Notch receptor cleavage and were then stimulated with growth medium or 3% FCS. DAPT treatment significantly decreased basal *Hey1* and *Hey2* mRNA levels. However, the addition of growth medium or 3% FCS still led to a considerable induction of *Hey* gene expression irrespectively if DAPT was present or not (**Fig. 2A**). This indicated that induction of *Hey* gene expression was independent of Notch receptor cleavage.

We repeated this experiment with a different kind of Notch inhibitor. Dominant negative Mastermind-like 1 protein (dnMAML) is a well-established tool to interfere with the nuclear Notch protein complex at the promoter of target genes.[21] dnMAML1 forms an inactive complex with NICD to inhibit the transcription of direct Notch target genes like *Hey1* and *Hey2* without affecting Rbp-Jk repressor function. Adenoviral transduction of HUVEC with dnMAML reduced the amount of *Hey1* and *Hey2* mRNA. However, dnMAML1 could not prevent the induction of *Hey2* transcription by serum, whereas the induction of *Hey1* expression was inhibited but not prevented by dnMAML1 (**Fig. 2B**).

**Fig 3:**
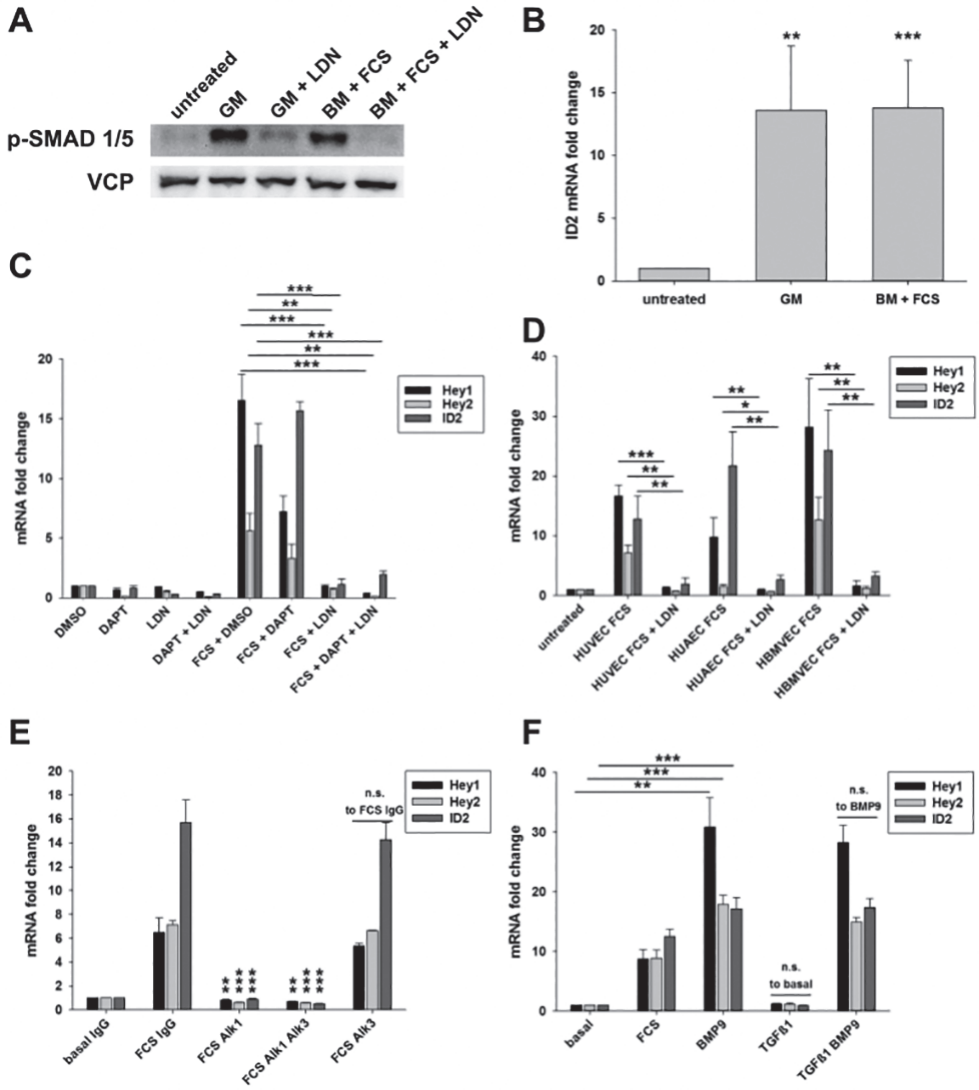
Transcriptional activation of *Hey1* and *Hey2* by serum is caused by BMP-Smad-signaling. A) HUVEC were cultured in growth medium and were pretreated for 30 min with 3μM LDN193189 or a corresponding volume of DMSO. The medium was left on the cells or it was replaced with fresh growth medium (GM) or basal medium (BM) with 3% FCS, both of which were containing LDN193189 or DMSO respectively. The western blot is showing phosphorylated Smad 1/5 (Ser463/465) and VCP in cells lysed 1.5h after medium change. B) HUVEC were cultured in growth medium, which was replaced with fresh growth medium or basal medium containing 3% FCS. Relative *ID2* mRNA levels are shown 1.5 h after medium change normalized to samples not subjected to medium change (untreated). C) HUVEC were cultured in growth medium and were pretreated over night with 25μM DAPT, 30 min with 3μM LDN193189 or the corresponding volumes of DMSO. Medium was not replaced (DMSO, DAPT, LDN, DAPT + LDN) or changed to basal medium with 3% FCS complemented with the inhibitors or DMSO respectively. The transcript levels of *Hey1*, *Hey2* and *ID2* were measured 1.5h after changing the medium. D) HUVEC, HUAEC and HBMVEC were cultured in growth medium and were pretreated 30 min with 3μM LDN193189 or DMSO. The medium remained unchanged (untreated) or was replaced with basal medium with 3% FCS containing LDN193189 or DMSO. The transcript levels of *Hey1*, *Hey2* and *ID2* were measured 1.5h after changing the medium. E) HUVEC were treated with medium containing no or 3% FCS and either soluble Alk1-Fc, Alk3-Fc or control IgG (5 μg/ml) for 1.5h. Transcript levels of *Hey1*,*Hey2* or *Id2* and significances are calculated to the basal medium IgG control. F) Cells were treated with basal medium containing 3% FCS, 10 ng/ml recombinant human BMP9 or 10 ng/ml recombinant human TGFβ1 for 1.5h. Transcript levels of *Hey1*, *Hey2* and *Id2* are calculated to the sample treated with basal medium. Mean and standard deviation, n≥3, * p<0.05, ** p<0.01, *** p<0.001.

The gold standard to detect Notch activity in endothelial cells is to detect the levels of cleaved and thus active Notch1 (NICD). The levels of NICD and Dll4 in HUVEC are low and difficult to detect with antibodies.[23] Therefore, we employed arterial endothelial cells (HUAEC), which have higher NICD levels. *Hey1* and *Hey2* expression was induced in these cells to a similar extent as in HUVEC (see below, and Fig. 3D). The addition of growth medium or 3% FCS did however only slightly increase the rate of Notch1 cleavage by approximately 15% (**Fig. 2C**).

To finally clarify this issue we analyzed the expression of other “classical” Notch target genes in endothelial cells upon treatment with growth medium or 3% FCS. *Hes1* expression was unaltered, while *Hes5*, *Dll4* and *ephrinB2* were only very slightly changed (**Fig. 2D**). This regulation fits quite well to the slightly increased cleavage rate of Notch1. However, the exaggerated *Hey1* and *Hey2* expression rates must be due to activation of another signaling pathway.

### BMP-Smad1/5 induces Hey1 and Hey2 gene expression in endothelial cells

It was shown that bone morphogenic protein signaling via Smad1/5/8 synergizes with Notch signaling to induce *Hey1* and *Hey2* transcription in endothelial cells,[11] and that BMP9 and BMP10 stimulate expression of Notch target genes.[14, 15] Since various BMPs are components of FCS,[15, 24] we tested BMP-Smad1/5 signaling as the potential inducer of *Hey1* and *Hey2* expression. Indeed, treatment of HUVEC with growth medium or 3% FCS led to a strong activation of Smad1/5 as determined by Western blotting with phospho-specific antibodies (**Fig. 3A**). The prototypical Smad1/5 target gene *Id2* was also induced more than 15-fold one and a half hours after treatment (**Fig. 3B**).

Dorsomorphin and LDN19318, a derivative of it, selectively inhibit BMP type I receptors such as Alk1, Alk2 and Alk3 with high affinity. [25, 26] LDN19318 abolished Smad1/5 phosphorylation upon treatment with serum (**Fig. 3A**). We pretreated HUVEC for 30 minutes with LDN193189 before the addition of fresh growth medium or 3% FCS. The inhibition of BMP-Smad1/5 signaling abrogated any induction of *Hey1*, *Hey2* or *Id2* (**Fig. 3C**). The same behavior was also observed in HUAEC and primary human microvascular brain endothelial cells (HBMVEC, **Fig. 3D**). This indicates that the induction of *Hey* gene expression in different primary endothelial cell cultures is mediated via BMP type I receptor signaling.

To further clarify, which of the BMP type I receptors is involved in the induction of *Hey*-genes by serum, we employed soluble, recombinant human Alk1 and Alk3 receptors fused to Fc. Blocking Alk3 signaling did not inhibit the induction of *Hey*-genes and Id2 by serum, whereas blocking Alk1 completely reversed these effects (**Fig. 3E**). Treating HUVEC with recombinant human BMP9, which is an activator of Alk1, did elicit transcription of *Hey1*, *Hey2* and *Id2* similar to serum (**Fig. 3F**) further confirming the role of Alk1. Alk1 can also be activated by TGFβ.[27] However, when treating HUVEC with recombinant human TGFβ1, there was no induction of *Hey1* and *Hey2* after 1.5 hours. Likewise, TGFβ1 could not further enhance the induction of *Hey* genes by BMP9 (Fig. 3F). These experiments show BMPs such as BMP9, which are components of serum, signal through Alk1 to induce transcription of *Hey1* and *Hey2*.

**Fig 4:**
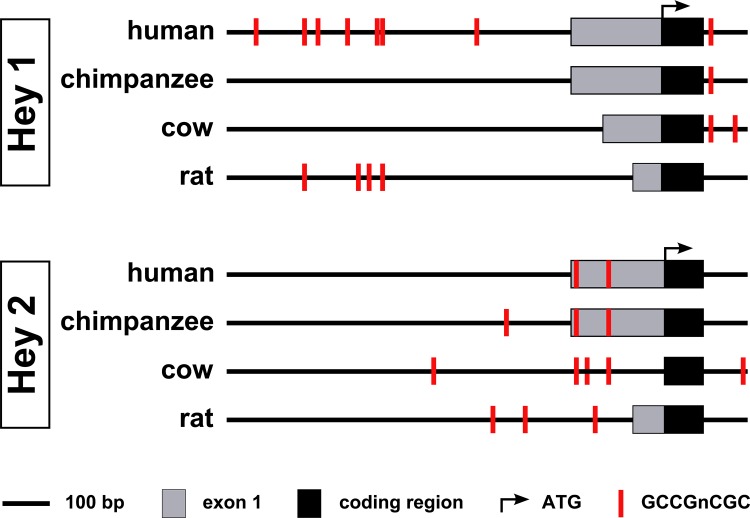
Smad1 binding sites in the promoter regions of *Hey1* and *Hey2*. Schematic alignment of the *Hey1* and *Hey2* promoters of *Homo sapiens* (human), *Pan troglodytes* (chimpanzee), *Bos Taurus* (cow) and *Rattus norvegicus* (rat). Potential Smad1 binding sites (GCCGnCGC) are depicted in red. For a clearer visualization, recognition sites, which are separated by less than 10 bp, are only depicted by one red bar.

Smad1/5 can activate the transcription of *Hey1* by binding to a cluster of GCCGnCGC motifs located within 1 kb upstream of the first exon.[28] *Hey1* and *Hey2* have redundant roles in endothelial cells and have comparably similar promoter regions. We analyzed the *Hey2* and *Hey1* promoters for such Smad1-binding motifs *in silico* (**Fig. 4**) and found two of these motifs in the proximal promoter of human *Hey2* (within 200bp upstream of the translational start site). These sites were conserved between several mammalian species, which indicates that these motifs have indeed a regulatory function and might be implicated in the induction of *Hey2* transcription by Smad signaling.

**Fig 5:**
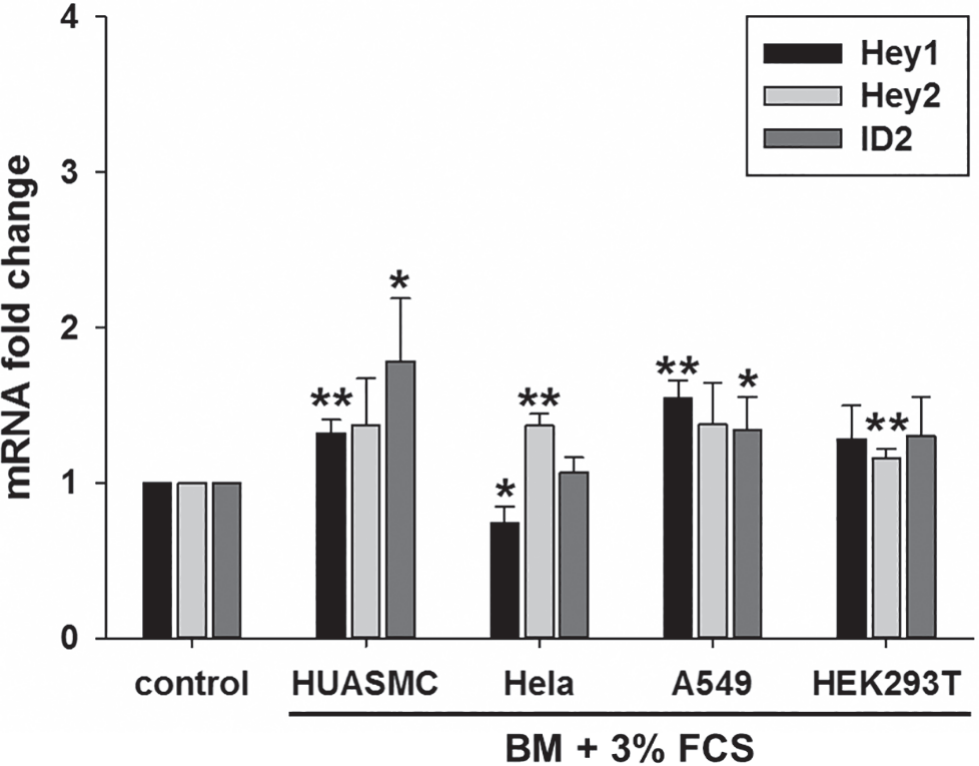
Marginal activation of *Hey1*, *Hey2* and *ID2* in non-endothelial cells. HUASMC, Hela, A549 and HEK293T cells were cultured in their standard medium, before they were left untreated or the medium was changed to basal medium containing 3% FCS. The transcript levels of *Hey1*, *Hey2* and *ID2* were determined 1.5h after changing the medium and were normalized to the samples, whose medium was not changed. Mean and standard deviation, n = 3, * p<0.05, ** p<0.01.

Lastly, we tested other cell types in the same experimental settings as described for endothelial cells. However, we could not observe such an induction of *Hey1*, *Hey2* or *Id2* in primary human vascular smooth muscle cells from the umbilical artery, HEK293, HeLa or A549 adenocarcinoma cells **(Fig. 5)**.This indicates that endothelial cells react much stronger to BMPs in the serum than other cell types and cell lines.

Taken together, the experiments revealed that bone morphogenic proteins such as BMP9 act through Alk1 on endothelial cells to stronglyinduce the expression of *Hey1* and *Hey2* genes in the first few hours upon medium exchange. Given that *Hey* gene induction is frequently used as readout for Notch activity, this should be carefully taken into account.

## Discussion

Induction of *Hey* gene transcription has been interpreted as activation of Notch signaling in hundreds of published studies. This work however suggests that careful analysis is necessary to draw such conclusions. We found that in particular endothelial cells react with a single wave of strong *Hey1* and *Hey2* expression to fresh serum. This wave lasted for three to four hours and was not dependent on Notch receptor cleavage but on Alk1 signaling. Therefore, this study revealed that bone morphogenic proteins (BMP) like BMP9, which are present in fetal serum, induce the expression of Hey genes, which play critical roles for endothelial cell biology.

The analysis of Notch signaling is complicated by the fact that four receptors and several ligands are present in the human genome.[3] One of the most reliable methods is the detection of cleaved Notch receptors and quantification of the intracellular domain (NICD).[9] However, this method relies on Western blotting and requires a substantial amount of cells. With immunocytochemistry one can detect nuclear NICD as an indicator of active Notch, however small differences between groups are difficult to measure. Alternatively, one can use reporter assays based on Rbp-Jk responsive DNA elements [29]. These have however the disadvantage that primary cells like HUVEC cannot be transfected in a sufficient degree. Therefore, the most convenient way to measure Notch activity in primary cells is the quantification of “Notch target genes”.

There is no doubt that *Hes* and *Hey* transcription factors are the primary target genes of Notch. However, one needs to consider that other signaling pathways may also control their expression. At least for Hes1 there is evidence that hedgehog, FGF, Wnt, and STAT signaling co-regulate transcription of the *Hes1* gene or the activity of the Hes1 protein.[11, 20, 30] In endothelial cells BMP-Smad cooperates with NICD to potentiate *Hes1* and *Hey1* expression.[12–15, 31] Our experiments showed that the gamma-secretase inhibitor DAPT which blocks Notch receptor cleavage was unable to interfere with serum-induced *Hey* gene induction in endothelial cells, whereas dominant-negative MAML1 (dnMAMAL1) inhibited the induction of *Hey1*. This may be due to the fact that BMP-Smad1/5 signaling acts through Rbp-Jk at the promoter elements of *Hes* and *Hey* genes.[12, 13, 32] dnMAML1 interferes with the Notch/Rbp-Jk complex,[21] and this may also disturb the action of Smad1/5 on Rbp-Jk. Taken together, the presented data suggest that the analysis of Notch activation in endothelial cells should be evaluated by the additional analysis of other Notch targets like *ephrinB2* and *Dll4*, which were not induced by serum,[23, 33] and if possible Western blotting against NICD.

This study revealed that *Hey* gene induction was dependent of Alk1 and the effects of serum could be phenocopied by supplying recombinant BMP9 to the cells. This protein activates Alk1 and is present in serum at comparably high concentrations.[15] Consistently, BMP9 was previously reported to induce *Hey1* expression in mesenchymal stem cells.[34]

*Hey1* and *Hey2* gene induction lasted only for about three hours after serum treatment. This may be due to the short half-life of bone morphogenic proteins, but also due to the fact that Hey1 and Hey2 proteins repress the expression of their own gene promoter.[34] Kagayama and colleagues have demonstrated nonsynchronized oscillatory gene expression of *Hes1* in several cell types.[18–20, 35] Synchronized cycling of *Hes* and *Hey* gene expression was also detected in the presomitic mesoderm in mice and zebrafish and this oscillatory gene expression is critical for the formation of somites.[30] In cell culture one can synchronize the cells by starvation followed by serum treatment. This leads to a three to four-fold gene induction of *Hes1* expression within 1–2 hours.[19] Fig. 5 shows that we observed such a weak wave of *Hey1* and *Hey2* gene expression in non-endothelial cells. However the induction by Alk1 signaling in endothelial cells was considerably stronger and thus probably masked this effect. Taken together, in the tested endothelial cells serum induces one large wave of *Hey* expression, whereas in other cell types serum seems to elicit a much weaker but oscillatory induction.[19, 20] The reason for this difference is unknown but could involve differential expression of BMP receptors or components of Smad signaling. For instance, the expression of *Alk1*, the receptor triggering the Hey induction described in this report, is mainly limited to endothelial cells.[36] The absence of Alk1 in non-endothelial cells would explain why serum fails to induce *Hey* expression in these systems.

We do not know if the presented findings have any implication for endothelial cells within an intact blood vessel. Nevertheless, BMP4, -9, and -10 circulate in high concentrations in serum,[15, 24] while BMP2, -4, -6, and -7 can be produced by injured or hypoxic tissue to promote angiogenesis.[11] As such we speculate that in situations like reperfusion of occluded vessels the contact with blood serum may stimulate a wave of Hey gene expression, which could have consequences for endothelial cell behavior, e.g. impaired sprouting angiogenesis.

## References

[1] Leimeister C, Externbrink A, Klamt B, Gessler M. Hey genes: a novel subfamily of hairy- and Enhancer of split related genes specifically expressed during mouse embryogenesis. Mechanisms of development. 1999;85(1–2):173–7. 10415358 10.1016/s0925-4773(99)00080-5

[2] Heisig J, Weber D, Englberger E, Winkler A, Kneitz S, Sung WK, et al Target gene analysis by microarrays and chromatin immunoprecipitation identifies HEY proteins as highly redundant bHLH repressors. PLoS genetics. 2012;8(5):e1002728 10.1371/journal.pgen.1002728 22615585 PMC3355086

[3] Kopan R, Ilagan MX. The canonical Notch signaling pathway: unfolding the activation mechanism. Cell. 2009;137(2):216–33. 10.1016/j.cell.2009.03.045 19379690 PMC2827930

[4] Fischer A, Gessler M. Delta-Notch—and then? Protein interactions and proposed modes of repression by Hes and Hey bHLH factors. Nucleic Acids Res. 2007;35(14):4583–96. 17586813 10.1093/nar/gkm477 PMC1950541

[5] Wiese C, Heisig J, Gessler M. Hey bHLH factors in cardiovascular development. Pediatric cardiology. 2010;31(3):363–70. 10.1007/s00246-009-9609-9 20033145

[6] Guruharsha KG, Kankel MW, Artavanis-Tsakonas S. The Notch signalling system: recent insights into the complexity of a conserved pathway. Nature reviews Genetics. 2012;13(9):654–66. 10.1038/nrg3272 22868267 PMC4369923

[7] Ntziachristos P, Lim JS, Sage J, Aifantis I. From fly wings to targeted cancer therapies: a centennial for notch signaling. Cancer Cell. 2014;25(3):318–34. 10.1016/j.ccr.2014.02.018 24651013 PMC4040351

[8] Penton AL, Leonard LD, Spinner NB. Notch signaling in human development and disease. Seminars in cell & developmental biology. 2012;23(4):450–7. 22306179 10.1016/j.semcdb.2012.01.010 PMC3638987

[9] Nakajima M, Shimizu T, Shirasawa T. Notch-1 activation by familial Alzheimer's disease (FAD)-linked mutant forms of presenilin-1. Journal of neuroscience research. 2000;62(2):311–7. 11020224 10.1002/1097-4547(20001015)62:2<311::AID-JNR16>3.0.CO;2-G

[10] Fischer A, Schumacher N, Maier M, Sendtner M, Gessler M. The Notch target genes Hey1 and Hey2 are required for embryonic vascular development. Genes Dev. 2004;18(8):901–11. 15107403 10.1101/gad.291004 PMC395849

[11] Beets K, Huylebroeck D, Moya IM, Umans L, Zwijsen A. Robustness in angiogenesis: Notch and BMP shaping waves. Trends in genetics: TIG. 2013;29(3):140–9. 10.1016/j.tig.2012.11.008 23279848

[12] Itoh F, Itoh S, Goumans MJ, Valdimarsdottir G, Iso T, Dotto GP, et al Synergy and antagonism between Notch and BMP receptor signaling pathways in endothelial cells. EMBO J. 2004;23(3):541–51. 14739937 10.1038/sj.emboj.7600065 PMC1271801

[13] Moya IM, Umans L, Maas E, Pereira PN, Beets K, Francis A, et al Stalk cell phenotype depends on integration of Notch and Smad1/5 signaling cascades. Dev Cell. 2012;22(3):501–14. 10.1016/j.devcel.2012.01.007 22364862 PMC4544746

[14] Larrivee B, Prahst C, Gordon E, del Toro R, Mathivet T, Duarte A, et al ALK1 signaling inhibits angiogenesis by cooperating with the Notch pathway. Dev Cell. 2012;22(3):489–500. 10.1016/j.devcel.2012.02.005 22421041 PMC4047762

[15] Ricard N, Ciais D, Levet S, Subileau M, Mallet C, Zimmers TA, et al BMP9 and BMP10 are critical for postnatal retinal vascular remodeling. Blood. 2012;119(25):6162–71. 10.1182/blood-2012-01-407593 22566602 PMC3383024

[16] Jakobsson L, Franco CA, Bentley K, Collins RT, Ponsioen B, Aspalter IM, et al Endothelial cells dynamically compete for the tip cell position during angiogenic sprouting. Nat Cell Biol. 2010;12(10):943–53. 10.1038/ncb2103 20871601

[17] Leimeister C, Dale K, Fischer A, Klamt B, Hrabe de Angelis M, Radtke F, et al Oscillating expression of c-Hey2 in the presomitic mesoderm suggests that the segmentation clock may use combinatorial signaling through multiple interacting bHLH factors. Dev Biol. 2000;227(1):91–103. 11076679 10.1006/dbio.2000.9884

[18] Imayoshi I, Isomura A, Harima Y, Kawaguchi K, Kori H, Miyachi H, et al Oscillatory control of factors determining multipotency and fate in mouse neural progenitors. Science. 2013;342(6163):1203–8. 10.1126/science.1242366 24179156

[19] Hirata H, Yoshiura S, Ohtsuka T, Bessho Y, Harada T, Yoshikawa K, et al Oscillatory expression of the bHLH factor Hes1 regulated by a negative feedback loop. Science. 2002;298(5594):840–3. 12399594 10.1126/science.1074560

[20] Yoshiura S, Ohtsuka T, Takenaka Y, Nagahara H, Yoshikawa K, Kageyama R. Ultradian oscillations of Stat, Smad, and Hes1 expression in response to serum. Proc Natl Acad Sci U S A. 2007;104(27):11292–7. 17592117 10.1073/pnas.0701837104 PMC1899192

[21] Weng AP, Nam Y, Wolfe MS, Pear WS, Griffin JD, Blacklow SC, et al Growth suppression of pre-T acute lymphoblastic leukemia cells by inhibition of notch signaling. Mol Cell Biol. 2003;23(2):655–64. 12509463 10.1128/MCB.23.2.655-664.2003 PMC151540

[22] Phng LK, Gerhardt H. Angiogenesis: a team effort coordinated by notch. Dev Cell. 2009;16(2):196–208. 10.1016/j.devcel.2009.01.015 19217422

[23] Adam MG, Berger C, Feldner A, Yang WJ, Wustehube-Lausch J, Herberich SE, et al Synaptojanin-2 binding protein stabilizes the Notch ligands DLL1 and DLL4 and inhibits sprouting angiogenesis. Circ Res. 2013;113(11):1206–18. 10.1161/CIRCRESAHA.113.301686 24025447

[24] Herrera B, Inman GJ. A rapid and sensitive bioassay for the simultaneous measurement of multiple bone morphogenetic proteins. Identification and quantification of BMP4, BMP6 and BMP9 in bovine and human serum. BMC cell biology. 2009;10:20 10.1186/1471-2121-10-20 19298647 PMC2663541

[25] Yu PB, Deng DY, Lai CS, Hong CC, Cuny GD, Bouxsein ML, et al BMP type I receptor inhibition reduces heterotopic [corrected] ossification. Nat Med. 2008;14(12):1363–9. 10.1038/nm.1888 19029982 PMC2846458

[26] Wrighton KH, Lin X, Yu PB, Feng XH. Transforming Growth Factor {beta} Can Stimulate Smad1 Phosphorylation Independently of Bone Morphogenic Protein Receptors. J Biol Chem. 2009;284(15):9755–63. 10.1074/jbc.M809223200 19224917 PMC2665096

[27] Oh SP, Seki T, Goss KA, Imamura T, Yi Y, Donahoe PK, et al Activin receptor-like kinase 1 modulates transforming growth factor-beta 1 signaling in the regulation of angiogenesis. Proc Natl Acad Sci U S A. 2000;97(6):2626–31. 10716993 10.1073/pnas.97.6.2626 PMC15979

[28] Dahlqvist C, Blokzijl A, Chapman G, Falk A, Dannaeus K, Ibanez CF, et al Functional Notch signaling is required for BMP4-induced inhibition of myogenic differentiation. Development. 2003;130(24):6089–99. 14597575 10.1242/dev.00834

[29] Lu FM, Lux SE. Constitutively active human Notch1 binds to the transcription factor CBF1 and stimulates transcription through a promoter containing a CBF1-responsive element. Proc Natl Acad Sci U S A. 1996;93(11):5663–7. 8643633 10.1073/pnas.93.11.5663 PMC39305

[30] Pourquie O. Vertebrate segmentation: from cyclic gene networks to scoliosis. Cell. 2011;145(5):650–63. 10.1016/j.cell.2011.05.011 21620133 PMC3164975

[31] Li F, Lan Y, Wang Y, Wang J, Yang G, Meng F, et al Endothelial Smad4 maintains cerebrovascular integrity by activating N-cadherin through cooperation with Notch. Dev Cell. 2011;20(3):291–302. 10.1016/j.devcel.2011.01.011 21397841

[32] Morikawa M, Koinuma D, Tsutsumi S, Vasilaki E, Kanki Y, Heldin CH, et al ChIP-seq reveals cell type-specific binding patterns of BMP-specific Smads and a novel binding motif. Nucleic Acids Res. 2011;39(20):8712–27. 10.1093/nar/gkr572 21764776 PMC3203580

[33] Iso T, Maeno T, Oike Y, Yamazaki M, Doi H, Arai M, et al Dll4-selective Notch signaling induces ephrinB2 gene expression in endothelial cells. Biochem Biophys Res Commun. 2006;341(3):708–14. 16430858 10.1016/j.bbrc.2006.01.020

[34] Nakagawa O, McFadden DG, Nakagawa M, Yanagisawa H, Hu T, Srivastava D, et al Members of the HRT family of basic helix-loop-helix proteins act as transcriptional repressors downstream of Notch signaling. Proc Natl Acad Sci U S A. 2000;97(25):13655–60. 11095750 10.1073/pnas.250485597 PMC17631

[35] Kobayashi T, Mizuno H, Imayoshi I, Furusawa C, Shirahige K, Kageyama R. The cyclic gene Hes1 contributes to diverse differentiation responses of embryonic stem cells. Genes Dev. 2009;23(16):1870–5. 10.1101/gad.1823109 19684110 PMC2725939

[36] Miyazono K, Kamiya Y, Morikawa M. Bone morphogenetic protein receptors and signal transduction. Journal of biochemistry. 2010;147(1):35–51. 10.1093/jb/mvp148 19762341

